# Traditional Farming Lifestyle in Old Older Mennonites Modulates Human Milk Composition

**DOI:** 10.3389/fimmu.2021.741513

**Published:** 2021-10-11

**Authors:** Antti E. Seppo, Rakin Choudhury, Catherine Pizzarello, Rohith Palli, Sade Fridy, Puja Sood Rajani, Jessica Stern, Camille Martina, Chloe Yonemitsu, Lars Bode, Kevin Bu, Sabrina Tamburini, Enrica Piras, David S. Wallach, Maria Allen, R. John Looney, Jose C. Clemente, Juilee Thakar, Kirsi M. Järvinen

**Affiliations:** ^1^ Division of Allergy and Immunology and Center for Food Allergy, Department of Pediatrics, University of Rochester School of Medicine and Dentistry and Golisano Children’s Hospital, Rochester, NY, United States; ^2^ Department of Microbiology and Immunology and Department of Biostatistics and Computational Biology, University of Rochester School of Medicine and Dentistry, Rochester, NY, United States; ^3^ Medical Scientist Training Program, University of Rochester School of Medicine and Dentistry, Rochester, NY, United States; ^4^ Department of Public Health Sciences & Environmental Medicine, University of Rochester School of Medicine and Dentistry, Rochester, NY, United States; ^5^ Division of Neonatology and Division of Gastroenterology, Hepatology and Nutrition, Department of Pediatrics, University of California, San Diego, La Jolla, CA, United States; ^6^ Mother-Milk-Infant Center of Research Excellence (MOMI CORE), University of California, San Diego, La Jolla, CA, United States; ^7^ Department of Genetics and Genomic Sciences, Icahn Institute for Data Science and Genomic Technology, Precision Immunology Institue, Icahn School of Medicine at Mount Sinai, New York, New York, NY, United States; ^8^ Division of Allergy, Immunology, and Rheumatology, Department of Medicine, University of Rochester School of Medicine and Dentistry, Rochester, NY, United States

**Keywords:** human milk, infants, allergy, oligosaccharide, cytokines, IgA, lifestyle, farming

## Abstract

**Background:**

In addition to farming exposures in childhood, maternal farming exposures provide strong protection against allergic disease in their children; however, the effect of farming lifestyle on human milk (HM) composition is unknown.

**Objective:**

This study aims to characterize the maternal immune effects of Old Order Mennonite (OOM) traditional farming lifestyle when compared with Rochester (ROC) families at higher risk for asthma and allergic diseases using HM as a proxy.

**Methods:**

HM samples collected at median 2 months of lactation from 52 OOM and 29 ROC mothers were assayed for IgA_1_ and IgA_2_ antibodies, cytokines, endotoxin, HM oligosaccharides (HMOs), and targeted fatty acid (FA) metabolites. Development of early childhood atopic diseases in children by 3 years of age was assessed. In addition to group comparisons, systems level network analysis was performed to identify communities of multiple HM factors in ROC and OOM lifestyle.

**Results:**

HM contains IgA_1_ and IgA_2_ antibodies broadly recognizing food, inhalant, and bacterial antigens. OOM HM has significantly higher levels of IgA to peanut, ovalbumin, dust mites, and *Streptococcus equii* as well TGF-β2, and IFN-λ3. A strong correlation occurred between maternal antibiotic use and levels of several HMOs. Path-based analysis of HMOs shows lower activity in the path involving lactoneohexaose (LNH) in the OOM as well as higher levels of lacto-*N*-neotetraose (LNnT) and two long-chain FAs C-18OH (stearic acid) and C-23OH (tricosanoic acid) compared with Rochester HM. OOM and Rochester milk formed five different clusters, e.g., butyrate production was associated with *Prevotellaceae*, *Veillonellaceae*, and *Micrococcaceae* cluster. Development of atopic disease in early childhood was more common in Rochester and associated with lower levels of total IgA, IgA_2_ to dust mite, as well as of TSLP.

**Conclusion:**

Traditional, agrarian lifestyle, and antibiotic use are strong regulators of maternally derived immune and metabolic factors, which may have downstream implications for postnatal developmental programming of infant’s gut microbiome and immune system.

## Introduction

Asthma and atopic diseases including food allergy, eczema, and rhinoconjunctivitis have increased exponentially over recent decades of industrialization and urbanization and are now the most common chronic medical conditions affecting children in the USA and are a global public health concern (1). Although heredity is a strong determinant of an allergic constitution, environmental factors, including a Western lifestyle, microbial exposures, and diet likely play a role in the rise of atopic diseases, which are characterized by Th2 responses. Cumulative data to date suggest that living on farms is associated with a major reduction in the risk of asthma and atopic diseases, including studies from Europe (2–4) and North America among the Amish (5, 6) and the Old Order Mennonites (OOM) (7–9). The individual factors that appear to be associated with this “farm-life effect” include consumption of unpasteurized farm milk (2, 10, 11) and exposure to farm animals, stables (2, 3) common in traditional one-family farms, but not in communal farms (6).

It is less appreciated that the temporal window for the immunomodulatory effects of a farming lifestyle extends from early postnatal to the prenatal period. Maternal microbial exposures during pregnancy have a strong and independent protective impact on atopic sensitization and allergic disease in their children (2–4, 12). This protection was associated with maternal exposure to farm animals and unboiled farm milk (3, 10), increased T regulatory cells (Tregs) in cord blood (13), and altered expression of receptors of the innate immunity (TLR2, TLR4, CD14) in school-aged children (3). These data suggest a maternal farmlife effect on protection against allergic diseases, but to date, only a single study has assessed the maternal, postnatal farming lifestyle effect related to human milk composition. In the European “Protection against Allergy-Study in Rural Environments” (PASTURE) cohort, IgA levels were higher in the milk of mothers with contact to farm animals and cats than in those unexposed during pregnancy (14).

Human milk contains immunologically active factors such as immune cells, cytokines, chemokines and hormones, immunoglobulins, critical growth factors, active enzymes including peroxidases and lysozymes, lactoferrin, saturated fatty acids, poly-unsaturated fatty acids (PUFAs) and additional secretory components, soluble CD14, TLR2, and tumor necrosis factor (TNF) receptor, along with maternal diet-derived food antigens (15). The role for maternal milk components in regulation of breastfed immunity is best demonstrated in mouse models, in which secretory breast milk IgA plays a role in the longterm gut homeostasis by regulating microbiota and host gene expression (16), and IgG immune complexes are shown to induce food-specific tolerance in offspring (17). Transforming growth factor (TGF)-β in murine milk plays a role in development of tolerance in the offspring (18). Epidermal growth factor (EGF) not only prevents dissemination of a gut-resident pathogen by inhibiting goblet cell-mediated bacterial translocation (19) but may also prevent food allergen uptake during breastfeeding, which may have implication for development of tolerance (20). Using human samples and infant cohorts, we have shown that human milk IgA controls excessive intestinal uptake of food antigens, which is associated with protection against food allergies in the offspring (21), as are higher levels of TGF-β1, along with IL-10 and IL-6 (22). In addition to providing immunostimulatory factors for the breastfed infant, breastfeeding significantly influences the development of the infant microbiome composition (23, 24), which influences gut dysbiosis in infancy. Early childhood precedes the development of multisensitization and atopic disease (25), including food allergy (26, 27) and asthma (28). One way breastfeeding impacts infant’s gut microbiota composition includes human milk oligosaccharides (HMOs), complex glycans that provide the main substrate for infant’s gut microbiota, particularly for Bifidobacteria and some Bacteroides (29, 30), and HMO compositional differences were detected in children protected against food allergy (31–33) and IgE-associated eczema (34). In addition, human milk contains microbiota, but the data are conflicting to which extent human milk may act as a source of bacterial species that colonize the infant gut (35–41). Human milk is also a rich source of short-chain fatty acids (SCFAs) (42), the function of which is unknown.

Breastfeeding provides continued exposure to the mother’s immune system during the crucial window of the first few months of life, when the infant’s immune system is continuously developing. However, the immunomodulatory composition of human milk characterized so far demonstrates a great deal of variability between mothers, which implies that the benefit of breastfeeding to the breastfed infants may differ between dyads. For the first time, we sought to characterize the differences in the multiple maternally derived immune and metabolic factors in human milk, including a broad characterization of specificity of IgA_1_ and IgA_2_, comparing farming and nonfarming lifestyles. Specifically, we here use the OOM as a model population of a lifestyle dating back 100 years. We hypothesized that because their lifestyle includes exposure to farm animals, homegrown food and unpasteurized milk, home births, and large family sizes and is limited in exposure to modern commodities such as cars and use of antibiotics, their human milk would have a different composition. Assessment of human milk composition can serve as a proxy for maternal immunity, the impact of which on allergic sensitization in the offspring starts in utero and expands postnatally through breastfeeding.

## Methods

### Study Population

For this cross-sectional study, we recruited a cohort of infants at low risk and those at high risk for allergies, as described before (41). The low-risk OOM of the Western NY reside in Penn Yan 65 mi southeast of Rochester and were recruited by a nurse midwife among prenatal visits in her clinic. Rochester urban and suburban mothers were recruited prenatally from the University of Rochester Medical Center (URMC) and Rochester, NY Obstetric practices. The OOM are a branch of the Mennonite Protestant tradition and of Swiss-German ancestry. They have a lifestyle incorporating growing up on a farm, having large families, exposure to numerous pets and farm animals, consumption of raw milk, low rate of smoking, antibiotic utilization, and infant vaccinations. They use a horse and buggy for transportation, grow most of their food, and many live on a dairy farm. OOM have very low rates of asthma, rhinitis, and eczema, e.g., the rate of asthma in OOM is only 1.8% compared with 12.0% in the general US population, by NHANES data (7). A follow-up questionnaire indicated that food allergies impact <2% of the OOM compared with 6.5% of the NHANES children (8). Only infants who were born full term (>36 weeks of gestation), with birth weight of >2,000 g, and generally healthy born to mothers without chronic infection, inflammatory disease, or known immunodeficiency or metabolic disease were included. Our protocol was approved by the Institutional Review Board of the University of Rochester Medical Center (RSRB52971).

**Figure 1:**
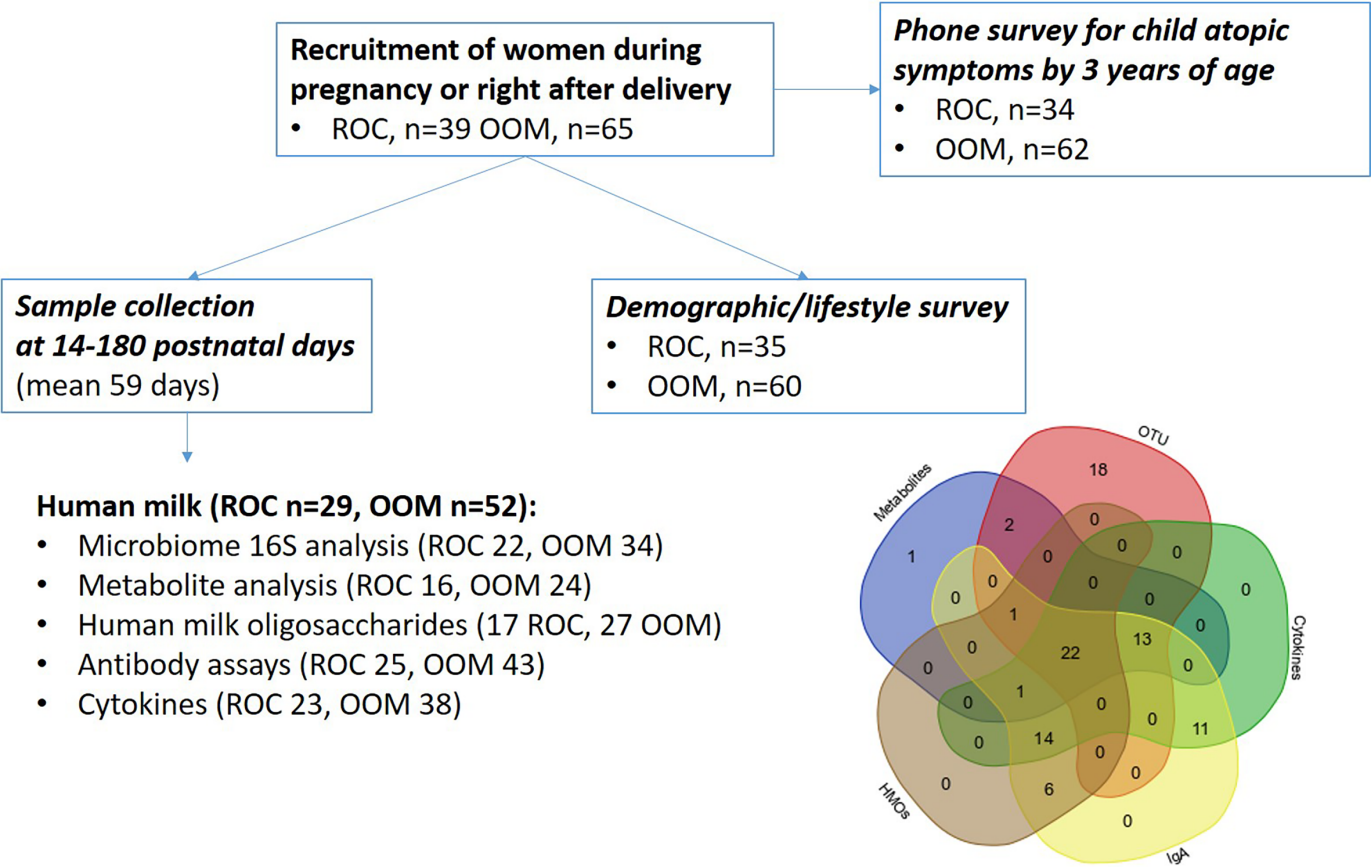
Schematic describing the cohort, samples, and assays of mother-infant pairs. OOM, Old Order Mennonite; ROC, Rochester.

Altogether, 65 OOM and 39 Rochester women were recruited pre- or immediately postnatally. Among them, human milk samples were received in 52 OOM and 29 Rochester mothers, collected between 2 weeks and 6 months of age. Details on study procedures and sampling are shown on **Figure 1**. The median duration of lactation at collection of human milk samples was 59 days (IQR 19).

### Follow-Up of Children for Atopic Symptoms

Presence of possible/likely atopic disease by the age of 3 years was defined through a telephone screening for possible allergic symptoms by a research coordinator, and a follow-up phone survey was performed by a pediatric allergist (KJ). We queried physician diagnosis not only of atopic dermatitis, allergic rhinitis, food allergy, and asthma but also symptoms consistent with atopic disease due to concerns of lower healthcare utilization by the OOM. The latter include chronic/remitting pruritic rash in a distribution age-typical for atopic eczema that had been treated by over-the-counter (OTC) or prescription steroidal creams or other anti-inflammatory topical treatments commonly indicated for atopic dermatitis; chronic or recurring symptoms of rhinorrhea/congestion/sneezing treated with antihistamines; symptoms suggestive of IgE-mediated food allergy and allergic proctocolitis; and recurrent wheezing episodes. Intermittent wheezing episodes associated with viral infections alone were not determined indicative of asthma due to young age.

### Maternal Prenatal Exposures

We collected data on maternal race and ethnicity, BMI, occupation, use of antibiotics during pregnancy, history of allergic diseases (asthma, eczema, allergic rhinitis, and food allergy), exposure to indoor and outdoor animals, consumption of unpasteurized milk and home-grown foods, the use of household cleaning agents, home heating and water sources and personal care products, and infant’s race and ethnicity, sex, mode, and place of birth, as well as the number of older siblings, initiation and duration of breastfeeding.

### Samples Collection and Processing

Human milk samples were collected in the morning, in UV-irradiated, sterile collection cups by sterile manual breast pumps (Harmony Breastpump, Medela) or by manual expression, wearing gloves. Foremilk was collected after cleaning the breast with Castille soap. All the samples were frozen at −20°C immediately upon collection until being transferred to −80°C within 4 weeks for long-term storage.

### Total and Specific IgA in Human Milk

*Total IgA* was measured using commercially available ELISA kit (Bethyl laboratories, Montgomery, TX, USA). *For food-specific IgA*, hen’s egg ovalbumin and bovine casein and β-lactoglobulin (BLG, Sigma, St. Loius, MO, USA) and defatted crude peanut extract were used as antigens. Microtiter plates (Nunc MaxiSorb, ThermoFisher Scientific, Waltham, MA, USA) were coated at a concentration of 20 µg/ml in phosphate-buffered saline (PBS) and incubated at 4°C overnight. After washing with PBS-0.05% Tween (PBS-T), plates were blocked with 1% bovine serum albumin (BSA) in PBS-T for 1 h at room temperature followed by washing with PBS-T. Human milk was diluted 1:5,000 for total IgA, 1:4 for specific IgA, and 1:2 for specific IgG. Purified human IgA (Bethyl Laboratories, Montgomery, TX, USA) was used as a standard curve for IgA and IgG. After washing, bound IgA antibody was detected using 1:100,000 diluted, HRP-conjugated goat-anti-human IgA for 1 h at room temperature. After washing, 100 µl of TMB substrate solution was applied to the plates, and the reaction was stopped and the optical density was measured at 450 nm.

To measure *aeroallergen-specific IgA* responses, we used a commercially available Luminex kit (Indoor Technologies, Charlottesville, VA, USA) including Alternaria mold, Bet v1 (birch), Phl p5 (timothy), Can f1 (dog), Mus m1 (mouse), Rat n1 (rat), Der p1 and Der f1 (dust mites), and Fel d1 (cat), which was modified for use as an IgA assay using secondary antibody with IgA specificity (Bethyl Laboratories, Mongomery, TX, USA).

To quantitate human milk IgA response to *select bacterial species*, we used whole bacteria (43) in a Luminex assay. Seven bacterial species were selected based on differential presence of IgA coating similarly as shown by Planer et al. (44), including gut commensals *Escheria coli*, *Enterobacter cloacae*, *Enterococcus faecalis*, *Morganella morganii*, *Lactobacillus reuteri*, *Salmonella enterica* spp. *typhimurium*, and *Streptococcus equii*, a horse respiratory pathogen. Bacterial species were grown in culture and used in whole bacteria ELISA to quantitatively measure specific IgA. Specificity was demonstrated by competitive inhibition of binding by the same bacteria and lack of competitive inhibition by other bacteria in the panel (data not shown) (**Supplementary Table 1**).

### Cytokines in Human Milk

Human milk cytokines were measured in milk supernatants by multiplex kits (Milliplex, Millipore, Billerica, MA, USA) and read on a Luminex 200 Multiplex analyzer (Luminex, Austin, TX, USA) after centrifugation and removal of fat layer as previously described (21). The following cytokines were measured: Th17 kit: interferon-gamma, IL-10, IL-12P70, IL-13, IL-17A, IL-9, IL-1β, IL-2, IL-23, IL-5, IL-6, IL-31, and Inflammation kit: IL-27, gp130/sIL-6Rβ, IL-34, IL-26, IL-19, IL-20, IL-29/IFN-Gamma1, IL-35, IL-32, BAFF/TNFSF13B, IL-11, APRIL/TNFSF13, IFN-β, sCD163, Pentraxin-3, LIGHT/TNFSF14, TSLP, sCD30/TNFRSF8, TWEAK/TNFSF12, Osteocalcin, IL-28A/IFN-Gamma2, sTNF-R2, Chitinase3-like 1, and sTNF-R1. TGF-β_1_, TGF-β_2,_ and TGF-β_3_ were measured using Bio-Plex TGF-β kit (Bio-Rad, Billerica, MA, USA) from samples acid activated to release the latent form (**Supplementary Table 1**).

### HMO Composition

Analysis of HMO composition and original data has been previously described by us (41). In brief, we measured the absolute concentrations of 19 most abundant HMOs (see **Supplementary Table 2**) by HPLC including type 1 and type 2 chains, branching, all types of fucosylation (α1-2, α1-3, and α1-4), which will allow determination of the mother’s Secretor and Lewis status, as well as all types of sialylation (α2-3 and terminal and internal α2-6).

### Human Milk Microbiome

Results and analysis of human milk and infant gut microbiota has been previously described (41, BioProject accession number PRJNA746977). In brief, genomic DNA from human milk samples was extracted using phenol-chloroform technique. Milk extraction was done using pelleted bacteria combined with fat layer in order to capture bacterial species that associate with fat globules and do not pellet during centrifugation. The V4 region of the 16S rRNA DNA gene was amplified in triplicate from all samples, pooled and sequenced on the Illumina MiSeq platform (paired-end 250bp) following standard protocols (45, 46), and data OTU level analyzed in previous publication was used (46).

### Human Milk Metabolites

Methods and original data have been previously published by us (41). In brief, human milk samples were analyzed for the concentrations of a comprehensive panel of 69 fatty acids (FAs) ranging in chain length from 2 to 26. The analysis was carried out at the Metabolic Invention Center (TMIC) at the University of Victoria, Alberta, Canada using a standard-based quantitation of 3-aminophenyl-derivatized FAs on UHPLC-MS/MS instrument (47). The data were manually annotated to Human Metabolome Database (HMDB) IDs. The data is available in the **Supplementary Table 3**.

### Human Milk Endotoxin

Endotoxin levels were measured by Limulus Amebocyte Lysate (LAL) chromogenic endotoxin quantification kit (Thermo Fisher Scientific) following manufacturer’s instructions.

### Statistical Analysis of Milk Factors

Cytokines and IgA were log10 transformed. The normality was tested using Shapiro-Wilk test for all HMOs, cytokines, short-chain fatty acids, and IgA levels. Rochester women and OOM were compared by *t*-test when Shapiro-Wilk test was not significant (*p* > 0.05) and Wilcoxon signed-rank test when Shapiro-Wilk test was significant (*p* < 0.05) in univariate analysis. Multivariate analysis was performed using generalized linear models with Gaussian error distribution when the Shapiro-Wilk test was nonsignificant and Quantile regression when the Shapiro-Wilk test was significant. The association among features were measured by Pearson’s correlation. All analyses were performed in R. To identify a differential activity of HMO cascades, CLIPPER was used (48).

Milk IgA, metabolites, microbiome, and HMOs were integrated, and differential network analysis was performed using xMWAS (49). For microbiome family level, OTU were used. Briefly, xMWAS constructs a matrix of pairwise correlation analysis using sparse partial least squares then ranks and filters the top association scores by *p*-value and association score. The edge lists are merged to a global network upon which community detection algorithms are utilized to group nodes into communities. Furthermore, centrality scores are calculated for each node in each group separately, then compared to generate differences between groups. The igraph package in R is used to generate a multidata integrative network (50). Community detection is performed using the multilevel community detection method (51).

## Results

### Maternal and Lifestyle Characteristics

Our questionnaires show OOM mothers have significantly lower rates of self-reported allergic rhinitis, atopic dermatitis, and food allergy and higher rates of exposure to farm animals, dogs, farm milk, and barns. They have higher rates of vaginal and home births and use of bleach and low rates of perinatal antibiotics and exposure to pesticides (**Table 1**). Previous report from this cohort reported some of the similar findings (41).

**Table 1:** Demographics and exposures of the study population.

	Rochester (*n* = 35)	OOM (*n* = 60)	*p*-Value
Age	31.9 (31.9, 32.0)	27.8 (27.8, 27.8)	<0.001
Body Mass Index	26.1 (26.0,26.1)	24.8 (24.8, 24.9)	0.41
Ethnidty/race			
Hispanic	5 (0.14)	0	0.0062
Not Hispanic	30 (0.86)	56 (1)	0.0062
Caucasian	24 (0.69)	56 (1)	<0.0001
African-American	4 (0.11)	0	0.0179
Asian	5 (0.14)	0	0.0062
Other	2 (0.06)	0	0.1391
n/a	0	4	
Atopic diseases			
Asthma	4 (0.13)	3 (0.05)	0.18
Hay Fever	14 (0.45)	10 (0.17)	0.004
Eczema	5 (0.16)	1 (0.02)	0.008
Food allergy	7 (0.23)	1 (0.02)	0.001
n/a	4	0	
Pregnancy delivery			
Vaginal delivery	25 (0.81)	59 (0.98)	0.007
Home Delivery	4 (0.13)	56 (0.93)	<0.001
Antibiotics	8 (0.26)	3 (0.05)	0.004
Occupation			
Housewife	6 (0.18)	13 (0.22)	0.792
Housewife/farmer	0	46 (0. 77)	<0.0001
Teacher	5 (0.15)	0	0.0046
Healthcare	8 (0.24)	0	0.0001
Other	14 (0.42)	1 (0.02)	<0.0001
n/a	2	0	
Exposures			
Cat	8 (0.26)	23 (0.40)	0.18
Dog	12 (0.39)	45 (0. 75)	0.001
Horse	3 (0.10)	57 (0.95)	<0.001
Cow	0	41 (0.68)	<0.001
Pig	0	6 (0.10)	0.0915
Poultry	0	38 (0.63)	<0.001
Rural home	0	56 (0.93)	<0.001
Farm milk	0	53 (0.88)	<0.001
Barn	0	40 (0.69)	<0.0001
n/a	4	0	
Cleaning agents			
Bleach	15 (0.45)	41 (0.68)	0.0458
Laundry detergent	32 (0.97)	57 (0.95)	
Antibacterial	21 (0.64)	34 (0.57)	0.6597
n/a	2	0	
Pesticides			
Insects (inside)	11 (0.18)	6 (0.18)	
Rodents (inside)	20 (0.33)	1 (0.03)	0.0006
Insects (outside)	41 (0.68)	9 (0.27)	0.0002
Rodents (outside)	16 (0.27)	1 (0.03)	0.0044
Weeds	48 (0.80)	10 (0.30)	<0.0001
n/a	2	0	

### Human Milk IgA Antibodies Differ Based on Lifestyle and Are Elevated in the OOM

**Figure 2:**
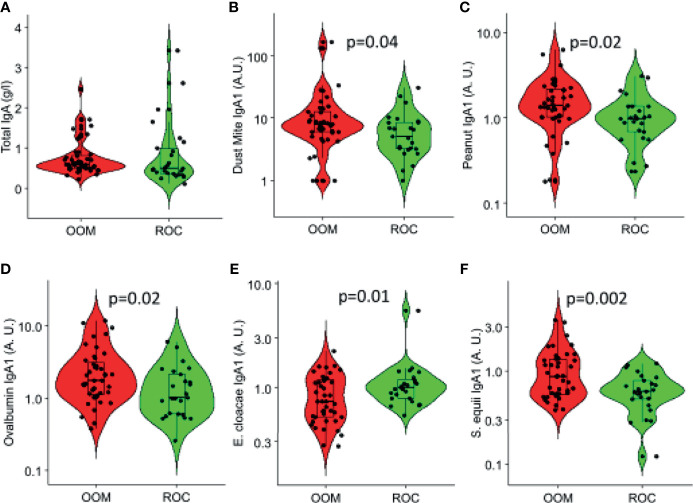
Total and specific IgA responses in human milk that are significantly different between the OOM (red) and Rochester (green) mothers. **(A)** Total IgA and IgA specific to **(B)** dust mite (Der p1 and Der f1), **(C)** peanut, **(D)** ovalbumin, **(E)** *E. cloacae*, and **(F)** *S. equii* are shown. Antigen-specific IgAs are normalized between 0 and 1.

Whereas, total IgA levels were comparable (**Figure 2A**) specific IgA_1_ and IgA_2_ levels were measured to nine common aeroallergens and three food antigens (milk, egg, and peanut). IgA_2_ was significantly elevated in OOM milk to dust mite (*p* = 0.04), peanut (*p* = 0.02), and egg ovalbumin (*p* = 0.02) (**Figures 2B–D**). Among nine bacterial species used in antibody assays, levels of IgA_1_ to *S. equis* were higher (*p* = 0.002) and those to *E. cloacae* were lower (*p* = 0.01) in the OOM compared with Rochester mothers’ milk (**Figures 2E, F**). Several other specific IgA antibodies appeared to be elevated in the OOM as well, but differences did not reach statistical significance.

**Figure 3:**
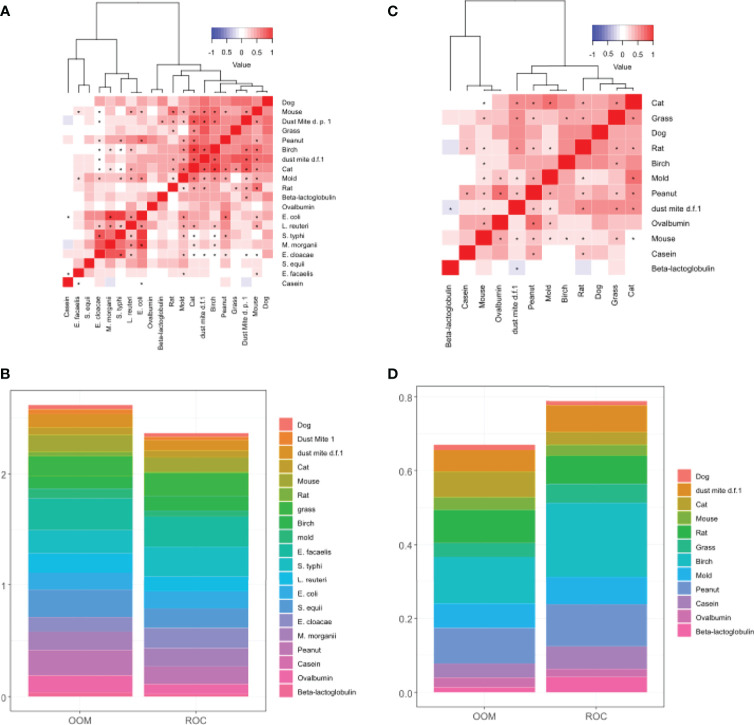
Characterization of IgA_1_ and IgA_2_ responses in human milk. Hierachial clustering of IgA_1_ levels depicting correlation between antigen-specific IgA1 **(A)** and IgA2 levels **(C)**. **(B)** Stacked bar plot of IgA_1_ levels normalized between 0 and 1 depicting higher accumulated levels of IgA_1_ in OOM. **(D)** Stacked bar plot of IgA_2_ levels normalized between 0 and 1 depicting higher accumulated levels of IgA_2_ in Rochester. IgA2 levels to bacteria antigens were too low to graph. D.p., *Dermatophagoides pteronyssinus*; d.f, *Dermatophagoides farina*. * indicates statistically significant correlation with p<0.001.

Comparison of cumulative levels of IgA_1_ and IgA_2_ across OOM and ROC indicated that IgA_1_ was expanded more in OOM (**Figure 3B**) whereas IgA_2_ was expanded more in Rochester (**Figure 3D**). Hierachial cluster analysis of IgA_1_ showed that aeroallergen-specific IgA_1_ antibodies all clustered together as did bacteria-specific IgA_1_ except for two bacteria (**Figure 3A**). Peanut-specific IgA_1_ clustered with aeroantigen IgA_1_ but the BLG-, OVA-, or casein-specific IgAs did not cluster with either one of the two clusters, neither did they cluster with each other. For IgA_2_, there was only one cluster, which included aeroallergen IgA_2_ and peanut, similar to IgA_1_ (**Figure 3C**). Food-specific IgA_2_ antibodies did not cluster with each similar to IgA_1_. Our initial results on a small number of samples showed that bacterial IgA_2_ was barely detectable, and therefore not further assessed.

In a multivariate analysis incorporating BMI, lifestyle, antibiotic use, age, atopy, cats, dogs, and parity, only casein-specific IgA_1_ remained significantly associated with lifestyle and was higher in Rochester mothers (**Table 2**). BLG-specific IgA_2_ was negatively associated with parity. IgA_1_ to cat and mouse were significantly negatively and positively associated with mother’s atopy, respectively. IgA_1_ to *S. equi* was significantly higher in cat owners.

**Table 2:** Multivariate analysis incorporating specific IgA levels, BMI, lifestyle, antibiotic use, age, atopy, cats, dogs, and parity.

	Lifestyle		Parity		BMI		Mother age (years)		Antibiotic use		Mother’s atopy		Cat in the household		Dog in the household	
	*p*-Value	*t*-Value	*p*-Value	*t*-Value	*p*-Value	*t*-Value	*p*-Value	*t*-Value	*p*-Value	*t*-Value	*p*-Value	*t*-Value	*p*-Value	*t*-Value	*p*-Value	*t*-Value
IgA																
β-lactoglobulin IgA2	0.401	−2.318	**0.006**	−0.859	0.809	−3.082	**0.048**	−0.245	0.118	2.104	0.674	−1.634	0.612	0.515	0.357	0.944
*S. equis* IgA1	0.174	−0.648	0.336	−1.393	0.794	−0.978	0.995	0.264	0.366	0.006	0.138	−0.918	**0.035**	2.210	0.075	1.847
Casein IgA1	**0.033**	1.739	0.218	2.246	0.080	1.261	0.315	−1.816	0.284	−1.024	0.787	1.093	0.562	0.588	0.074	1.861
Cytokines																
APRIL	0.478	−0.722	0.284	−1.099	0.873	−0.162	0.350	0.956	**0.033**	−2.278	0.229	−1.239	0.491	−0.701	0.519	−0.656
BAFF	0.760	−0.310	0.385	−0.887	0.628	0.492	0.827	0.221	0.547	−0.613	**0.041**	−2.175	0.952	0.061	0.831	−0.217
IFN-λ3	**0.038**	2.213	0.830	0.217	0.286	−1.095	0.528	−0.642	0.144	−1.518	0.542	−0.620	0.612	0.516	0.540	−0.623
sTNF.R1	**0.052**	−2.056	**0.014**	−2.672	0.480	0.719	0.065	1.950	**0.040**	−2.188	0.454	−0.763	0.526	−0.645	0.507	−0.674
TGF-β2	0.058	−2.001	**0.013**	−2.731	0.783	0.279	**0.052**	2.057	0.245	−1.197	0.294	−1.076	0.508	−0.674	0.861	0.178
Human milk oligasaccharides (HMOs)																
LNnT	0.403	−0.860	0.919	−0.104	0.423	−0.823	0.670	−0.435	**0.017**	−2.688	0.436	0.801	0.158	−1.487	0.773	−0.294
DFLac	0.208	1.314	0.249	1.198	0.103	1.738	0.289	−1.099	**0.044**	2.203	0.329	−1.010	0.184	−1.392	0.287	1.103
6.SL	**0.023**	−2.532	**0.015**	−2.759	0.129	1.607	0.302	1.069	0.780	−0.285	0.060	2.037	0.304	1.065	0.084	−1.848
LNFP.I	0.982	0.023	0.837	0.210	0.314	−1.042	0.221	−1.278	**0.031**	−2.386	0.196	1.353	0.331	1.006	0.498	0.695
LNFP.II	0.106	1.720	0.193	1.364	0.510	0.675	0.253	−1.188	**0.045**	2.188	0.742	−0.335	0.287	−1.103	0.810	−0.245
DSLNT	0.585	0.558	0.955	−0.057	0.243	−1.216	0.085	−1.843	0.473	−0.736	**0.030**	2.389	0.716	−0.371	0.684	−0.415

S. equis, Streptococcus equis. Bold font indicates p < 0.05.

### Characterization of Human Milk Cytokines Shows Higher Levels of IgA-Producing Cytokines in OOM

**Figure 4:**
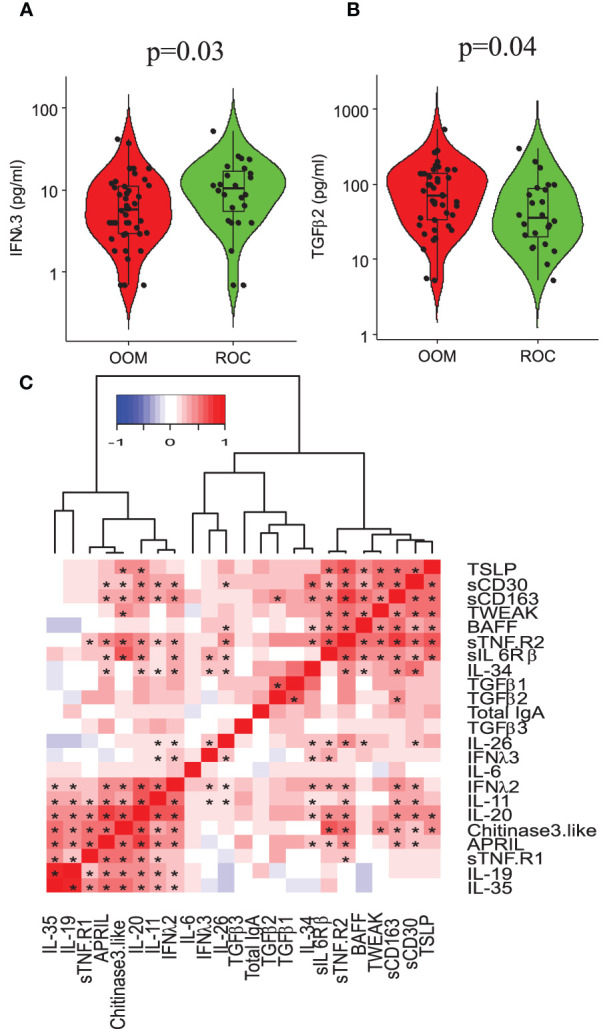
Cytokine profiling of human milk and its association with total IgA levels. Distributions of **(A)** IL-6 (*p* = 0.02, 0.05 by Wilcoxon signed-rank test) and **(B)** TGF-β2 (*p* = 0.05) in OOM and ROC population shown on logarithmic scale to the base 10. **(C)** Hierarchial cluster analysis showing correlation between a panel of cytokines. Asterisk, adjusting for mother’s atopy in multivariate analysis.

The levels of IFN-λ_3_ (*p* =0.03) and TGF-β_2_ (*p* = 0.04) were higher in the milk of OOM mothers compared with Rochester mothers (**Figures 4A, B**). In a multivariate analysis incorporating BMI, lifestyle, antibiotic use, age, atopy, cats, dogs, and parity, IFN-λ_3_ and sTNFR1 were found to be significantly higher and lower in OOM compared with Rochester, respectively (**Table 2**). sTNFR1 and TGF-β_2_ were also significantly lower with higher parity. In addition, functionally related cytokines showed similar trends suggesting biological mechanisms associated with lifestyle and other covariables. As an example, IL-6, TGF-β_1_, and TGF-β_2_, cytokines involved in IgA production, are grouped together along with other cytokines involved in IgA production including TSLP, APRIL, and TGF-β_3_ (**Figure 4C**). TWEAK and BAFF formed a separate cluster.

### Specific HMOs Are Correlated With Lifestyle and Maternal Use of Antibiotics

**Figure 5:**
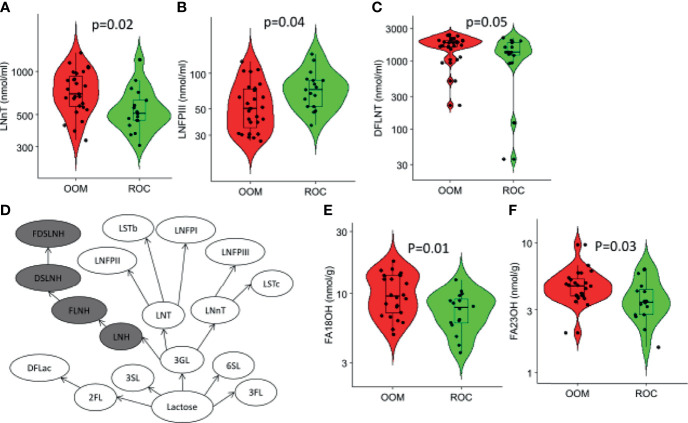
Characterization of human milk oligosaccharides (HMOs) and fatty acid metabolites that are different in Rochester and OOM population. Shown are the three HMOs between the OOM and Rochester mothers by the *t*-test **(A)** LNnT, **(B)** LNFPIII, and **(C)** DFLNT. **(D)** Path-based analysis was performed to find differences in the OOM *vs*. Rochester. The highlighted path has significantly lower (*p* = 6*e*−10) activity in OOM. The difference in the pathways is based on homoscedacity. In other words, the paths that have HMOs with different variance across two groups are selected. **(E, F)** Fatty acids that were significantly different between the OOM and Rochester.

Levels of LNnT and DFLNT were significantly elevated in milk of OOM mothers (*p* = 0.02 and *p* = 0.05, respectively), and levels of LNFPIII were higher in the milk of Rochester mothers (*p* = 0.04) (**Figures 5A–C**). In a multivariate analysis incorporating BMI, lifestyle, antibiotic use, age, atopy, cats, dogs, and parity, a strong correlation occurred between maternal antibiotic use and levels of several HMOs (**Table 2**). Moreover, after controlling for other variables, 6’SL was significantly higher in OOM compared with Rochester mothers. Finally, to investigate whether certain metabolic transitions are different between OOM and Rochester groups, we performed a topological analysis by CLIPPER using HMO biosynthetic pathway. The metabolic transitions starting from LNH to FDSLNH were significantly higher in Rochester mothers (**Figure 5D**).

### Characterization of Milk Metabolites Between Lifestyles

Characterization of short-, medium-, and long- chain fatty acids in human milk from OOM and Rochester communities revealed only two long-chain fatty acids both of which are elevated in OOM milk (**Figures 5E, F**). Those are C-18OH (stearic acid) and C-23OH (tricosanoic acid).

### Endotoxin

Endotoxin levels were undetectable in most samples, and there were no significant differences between the groups.

### Integration of Milk Microbiota With Milk Factors

**Figure 6:**
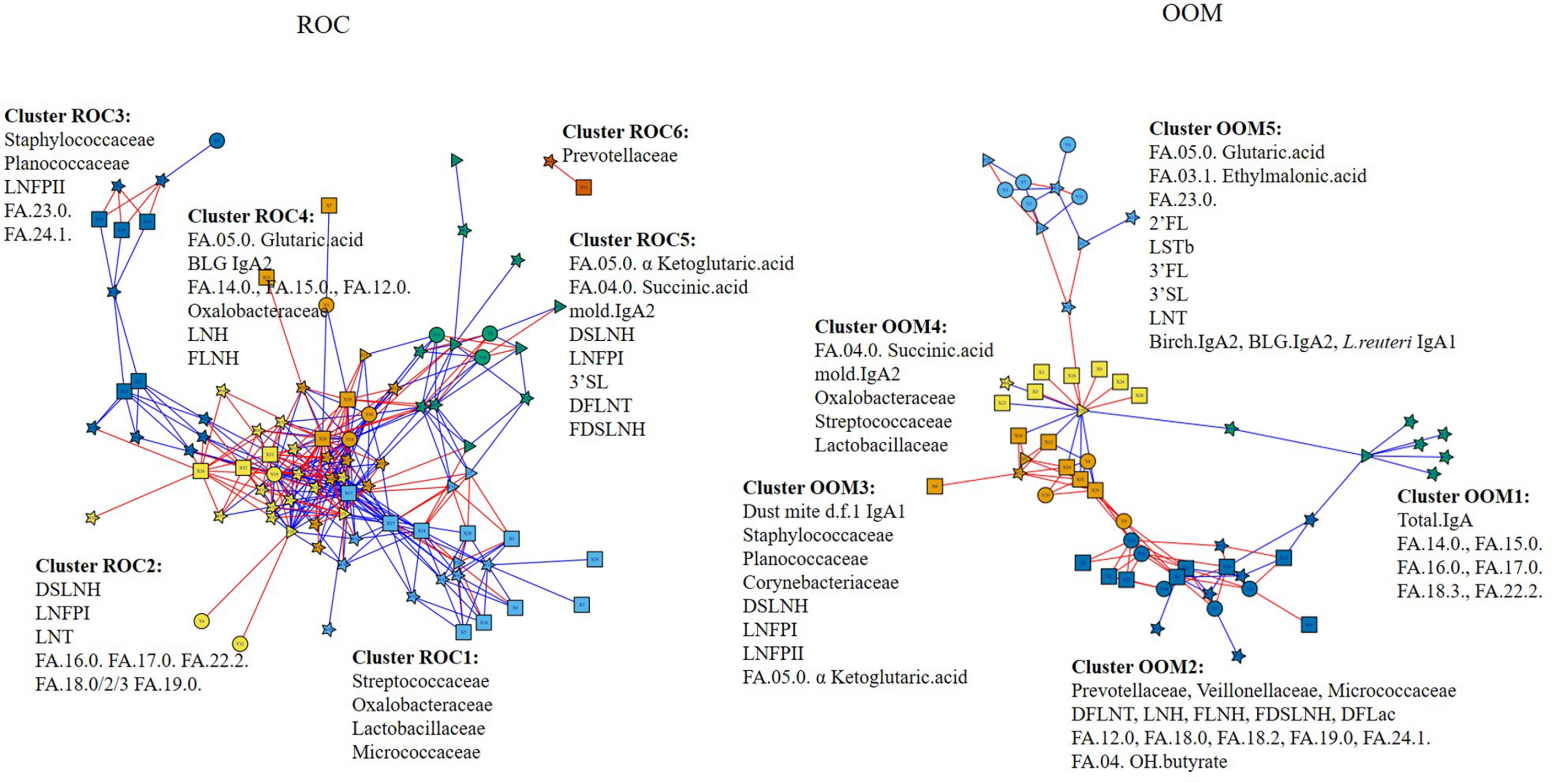
Integrated communities of human milk components in OOM and Rochester populations. Associations between HMOs, fatty acid metabolites, IgA, specific IgA, and milk microbiome were performed using partial least square regression to identify communities in eight Rochester mother (left) and 12 OOM mother (right) populations. Edges represent *R* ^2^ > 0.7 for ROC and 0.6 for OOM. Red and blue edges represent positive and negative correlations, respectively. The shapes of the nodes represent the following components: squares, human milk OTUs; circles, HMOs; triangles, IgA; and stars, fatty acids.

To identify milk factors associated with milk microbiota, we performed differential network analysis measuring associations across data types (**Figure 6**). OOM- and ROC-specific networks were identified when HMOs, IgA, and metabolites were combined with microbiota. Community detection was performed to identify topological modules composed of related biomolecules (52, 53). The number of communities are optimized as previously described (51, 54). OOM milk was characterized by five communities which we compare with Rochester milk communities. All associations described below are significant at *p* < 0.05. *Cluster OOM1:* total IgA was associated with several medium to long-chain fatty acids in OOM but not in Rochester milk. *Cluster OOM2:* previously identified milk microbiota Prevotellaceae, Veillonellaceae, and Micrococcaceae are grouped with several fatty acides including butyrate and HMOs including DFLNT, LNH, FLNH, FDSLNH, and DFLac in OOM. Butyrate was specifically positively correlated with highly abundant Micrococcaceae in OOM. Differentially abundant DFLNT was positively correlated with Micrococcaceae, Veillonellaceae, and Planococcaceae. Among these, only Micrococcaceae was found in Rochester communities but was not associated with HMOs and butyrate. *Cluster OOM3:* highly abundant Staphylococcaceae and LNFPII formed one community in both lifestyles but was also grouped with IgA1 specific to dust mite in OOM. Moreover, Staphylococcaceae was positively correlated with IgA_1_ to dust mite and mold in OOM. *Cluster OOM4:* IgA_2_ to mold was grouped with succinic acid in both lifestyles but was also clustered with Streptococcaceae and Lactobacillaceae in OOM. *Cluster OOM5:* only one cluster did not include any microbial taxa and was composed of fatty acids including glutaric acid and ethylmalonic acid among others, HMOs 2FL, LSTb, 3’FL, 3’SL, LNT, and IgA_2_ to birch and BLG and IgA_1_ to *L. reuteri*.

### Integration of HMOs With Infant Gut Microbiota

Although the number of samples were small, there were some interesting initial associations between human milk microbiota, HMOs, and infant gut microbiota. Especially, the association between Bifidobacteriaceae with LNT and LNFPII in Rochester, but between the same taxa and different HMOs, LNnT and LNFPIII in OOM infants, is of interest (**Supplementary Figure 1**).

### Association of Human Milk Components With Development of Atopic Disease

**Figure 7:**
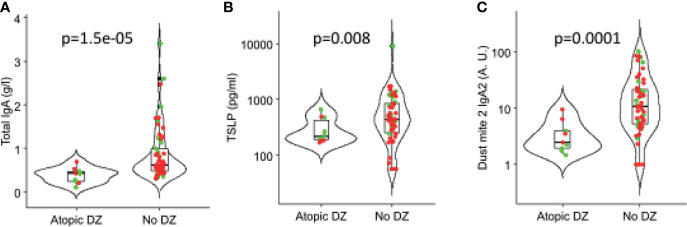
Association of IgA and cytokines in human milk with atopic outcomes in children in the first 3 years of life. **(A)** Total IgA, **(B)** TSLP (thymic stromal lymphopoietin), and **(C)** Dust mite 2 IgA2. Red: OOM. Green: Rochester children.

Among the many cytokines, TSLP (*p* = 0.002) was found in lower concentrations in milk of mothers with an infant with atopic disease (**Figure 7**). Also, total higher levels of IgA (*p* = 0.000015) and IgA_2_ to dust mite (*p* = 0.0001) were associated with protection against atopic diseases (**Figure 7** **)**.

## Discussion

To our knowledge, this is the first multicomponent characterization and network model of human milk composition spanning antibodies, cytokines, human milk oligosaccharides, metabolites, and gut microbiome. It is also the first of such characterization among mother-infant pairs with different lifestyles and risk for allergic diseases. We also performed the most comprehensive assessment of IgA_1_ and IgA_2_ antibodies to date. We show that OOM milk is more abundant in many immune components, especially those involved in IgA immunity, and LNnT which enhances *Bifidobacterium* enrichment. Maternal use of antibiotics was associated with differences in several HMO concentrations. These data support the concept that maternal lifestyle and antibiotic use are regulators of human milk composition, which may have downstream implications for postnatal developmental programming of infant’s gut microbiome and immune system. Considering that the OOM lifestyle dates back about a century, our data may also imply that there has been a change in human milk composition along with its protective properties over time. Although the function of all human milk components is not well known, many of them have downstream implications for postnatal developmental programming of infant’s gut microbiome and immune system suggesting potential benefit to infants born to the OOM community.

While the regulation of human milk composition of immune factors is poorly known, a few prior studies have documented differences in human milk IgA levels between mothers in different countries (14, 55, 56) and those avoiding or consuming particular foods (21), implying that maternal exposures impact milk composition. Consistent with an earlier farmlife study showing higher levels of IgA in farming lifestyle mothers’s milk, we showed upregulation of specific IgA antibodies in the OOM milk. Higher IgA response in the OOM milk was seen specifically to peanut, egg ovalbumin of hens, dust mite, and *S. equi*, a horse respiratory pathogen; whereas Rochester milk had higher IgA response only to a single antigen, namely *E. cloacae*, which is a gut commensal. These findings could be attributed to specific antigenic exposures between the OOM and Rochester mothers, for example, the higher levels of egg-specific IgA could reflect that eggs are a staple in their daily diet of the OOM who commonly keep chicken; however, elevated antibody levels against milk of cows or peanut are hardly due to more common exposures in the OOM. Furthermore, aeroallergen-specific IgA responses were elevated to a broad array of aeroallergens in the OOM (although only dust mite reached statistical significance), however, exposure to such inhalant antigens is unlikely to be more common in the OOM who as an example do not allow pets inside the home. These data may imply another mechanism, aside from higher exposures, boosting mucosal antibody production in the OOM. The ultimate function for these specific IgA antibodies is unknown, although we and others have previously reported that higher levels of IgA and specific IgA are associated with protection against milk allergy of cows in an infant (21, 57, 58). While mechanism of protection is unknown, IgA was shown by us not only to decrease the uptake of specific food antigens through the gut epithelium but also to enhance targeted antigen uptake to gut-associated lymphoid tissues in an antibody-dependent manner (21, 59). The presence or the role of aeroallergen- or commensal-specific IgA in human milk has not been previously assessed, although secretory IgA in breast milk plays a role in longterm gut homeostasis by regulating microbiota and host gene expression (16).

In this novel study, we report on the presence of both IgA_1_ and IgA_2_ to a broad selection of food, aeroantigens, and commensals. IgA_1_ antibodies appeared more commonly detectable than IgA_2_, and for example, commensal-specific IgA_2_ responses were undetectable. Our data are consistent with few previous studies suggesting that protein antigens such as BLG and tetanus toxoid predominantly induce IgA_1_; however, our data do not support a predominant IgA_2_ responses to polysaccharide antigens as reported with pneumococcal, meningococcal, and *Hemophilus influenzae* (60, 61). Clustering analyses showed that aeroallergens clustered together; however, they also clustered with peanut, which may be explained by the crossreactivity between peanut and pollen. Similarly, commensal IgA responses clustered together. Interestingly, milk and egg antigens were not clustered with either of the two larger clusters, neither were they clustered with each other. This may imply an exposure route that is different from that of the other antigens and from each other, such as the skin or a specific part of the GI tract based on the differential stability of these food antigens in the digestive tract.

The cytokines measured here are of interest because a few prior studies have documented differences in these human milk cytokine levels between geographic locations and countries. Higher levels of IL-10 were seen in Estonia mothers than in Swedish mother (55) and that of TGF-β1 in Finland compared with other European countries (14). Another study showed differences in cytokines and growth factor levels between developing and developed world countries (62, 63). Here, we found IFN-λ_3_ and TGF-β_2_ at higher levels in the OOM. Interestingly, a study performed in Sweden among mothers who had immigrated from a developing country early on had higher levels of IL-6, IL-8, and TGF-β_1_ in their milk suggesting that early life exposures have an impact on the immune system that sustains into adulthood and impacts human milk composition (64). In addition, we found several cytokines involved in IgA production clustered together, such as IL-6, TGF-β_1_, and TGF-β_2_, which clustered with TGF-β_3_ and APRIL and TSLP. This may indicate common pathways involved in cytokine production for the benefit of inducing upregulation of IgA levels in human milk. The cytokines measured here are of special interest, because we had previously identified those in higher concentrations associated with protection against food allergy (22). It may therefore be plausible that milk cytokines and IgA function in concert to support the infant against immune-mediated diseases.

Several large studies have confirmed the role of breastfeeding in modulating gut microbiome composition (24), showing that breast milk selects for intestinal microbiome dominated by *Bifidobacterium* for the first year of life (65). This is due to HMOs that are complex glycans and the third largest solid component in human milk. They are typically nondigestible by humans but provide the main substrate for infant’s gut microbiota, particularly, for *Bifidobacterium* and some *Bacteroides* (29, 30). Therefore, when breastfeeding ceases, signficant shifts in gut microbiome composition are seen with Clostridium and Bacteroides species replacing Lactobacilli-, Bifidobacteria-, and Enterobacteriaceae-dominated microbiota (66–68). We recently reported that HMO composition was related to maternal probiotic exposures (33). In the current report, we identified specific HMO levels associated with maternal antibiotics, suggesting a role for maternal microbiome in regulation of HMO composition. In this study, we found few differences in single HMOs between the OOM and Rochester mothers (LNnT, LFPIII, and DFLNT). However, because metabolic transitions between HMOs are not necessarily known, we sought to investigate whether certain conversions are different between OOM and Rochester groups. The path starting from LNH to FDSLNH were significantly higher in Rochester mothers; however, we found no specific HMO composition in relation to atopic disease development. This is in contrast with prior studies that have associated specific HMO profiles with atopic diseases (31–33), possibly explained by the small sample size.

Of particular interest in human milk is the presence of fatty acids, especially short chain fatty acids (SCFA), which are solely metabolized by gut bacteria from otherwise indigestible carbohydrates of fiber-rich diets. There is one known study so far indicating lower levels of acetate and butyrate in atopic mothers (42). SCFAs in human milk are likely produced by the maternal gut microbiota and distributed to the mammary gland *via* the circulation (42). We found an association between milk butyrate and specific taxa such as *Prevotella*, which is a known producer of butyrate (69), which may provide the first current evidence that SCFA in humans be produced by the human milk microbiota. Alternatively, maternal diet may contribute to SCFA levels in human milk. We also found higher levels of tricosanoic acid and palmitic acid in the OOM milk, but the implication of this unclear. FAs such as palmitic acid in human milk (70, 71) has been associated with changes in infant gut microbiota, but causality is unclear.

In this cohort, development of atopic disease in early childhood was more common in Rochester and associated with lower levels of IgA to dust mite as well as TSLP, but larger longitudinal studies are needed to conclusively assess whether OOM milk composition may provide benefit to their infants against development of allergic diseases. Assessing the relationships between human milk components and development of atopic outcomes in the children will only provide correlative data and causal relationship cannot be drawn. Human milk is only one of the many factors that can affect the development of atopic outcomes by 3 years of age. Not all the factors have been assessed or controlled for in this study. Further limitations include a small sample size of this pilot study and that all the assays were not performed on every sample due to limitation of volume. Allergic outcomes were limited to self-report of physician diagnosis or symptoms of atopic disease, although assessment was done by a pediatric allergist by telephone follow-up. Strenghts include access to a unique population living a traditional, agrarian lifestyle, with lifestyle representative of that commonly encountered a century ago, when allergic diseases were rare.

Boosting immunomodulatory properties of human milk might present an opportunity to modulate infant’s gut microbiome and risk for allergic diseases. Differences in human milk composition may have implications for developmental programming of the infant immune system postnatally by human milk but are also likely reflective of the maternal immune environment with implications to prenatal developmental programming. More studies are clearly needed to understand the role of maternally derived prenatal and postnatal factors on allergic diseases.

## Data Availability Statement

The datasets presented in this study can be found in online repositories and in **Supplementary Material**. The names of the repository/repositories and accession number(s) can be found in the article.

## Ethics Statement

The studies involving human participants were reviewed and approved by University of Rochester Institutional Review Board. Written informed consent to participate in this study was provided by the participants’ legal guardian/next of kin.

## Author Contributions

AS, JL, CM, and KJ conceived the overall study and supervised the research. AS, RC, CP, RP, SF, PR, JS, CY, LB, KB, ST, EP, DW, MA, RL, JC, JT, and KJ contributed to the literature search, data collection, and data analysis. AS, JT, JC, and KJ provided the figures and drafted the manuscript, with additional input from all authors. All authors contributed to the article and approved the submitted version.

## Funding

The project described was supported by Grant Numbers K08 AI091655 (KJ), U01 AI131344 from the National Institute of Infectious and Allergic Diseases (KJ), R21 TR002516 from the National Center for Advancing Translational Sciences, Founders’ Distinguished Professorship in Pediatric Allergy (KJ), and by Pilot Award (P30 ES001247) from Environmental Health Sciences Center (JL, CM), National Institute of Environmental Health Sciences, and Rochester CTSA award UL1 TR000042 from the National Center for Advancing Translational Sciences (JL, CM) of the National Institutes of Health. The content is solely the responsibility of the authors and does not necessarily represent the official views of the National Institutes of Health.

## Conflict of Interest

The authors declare that the research was conducted in the absence of any commercial or financial relationships that could be construed as a potential conflict of interest.

## Publisher’s Note

All claims expressed in this article are solely those of the authors and do not necessarily represent those of their affiliated organizations, or those of the publisher, the editors and the reviewers. Any product that may be evaluated in this article, or claim that may be made by its manufacturer, is not guaranteed or endorsed by the publisher.

## Supplementary Material

Supplementary Figure 1 Association between HMOs, human milk microbiome, and infant gut microbiome. Associations were measured using partial least square regression to identify communities represented by networks with blue, green, yellow, and gold node colors; using six Rochester mother-infant pairs and six OOM mother-infant pairs. Edges represent *R* ^2^ > 0.73 for ROC and 0.63 for OOM. Red and blue lines represent positive and negative correlations, respectively. The shapes of the nodes represent components: squares, human milk microbiota (underlined); circles, HMOs; triangles, infant stool microbiota.

Supplementary Table 1 Milk HMOs, IgA, and cytokines.

Supplementary Table 2 Human milk oligosaccharides measured.

Supplementary Table 3 Milk metabolites.

## Abbreviations

AUC, area under curve; BMI, body mass index; FA, fatty acid; qPCR, quantitative PCR; HMO, human milk oligosaccaharide; IQR, interquartile range; LefSe, linear discriminant analysis effect size; LNnT, lacto-*N*-neotetraose; OOM, Old Order Mennonite; OTU, operational taxonomic unit; PERMANOVA, permutational multivariate ANOVA; rRNA, ribosomal RNA; SCFA, short-chain fatty acid; Treg, regulatory T cell.
