# Supplementary figures and images for: Traditional Farming Lifestyle in Old Older Mennonites Modulates Human Milk Composition
Source: Front Immunol. 2021 Oct 11;12:741513. doi: 10.3389/fimmu.2021.741513 (PMC8545059; doi:10.3389/fimmu.2021.741513)

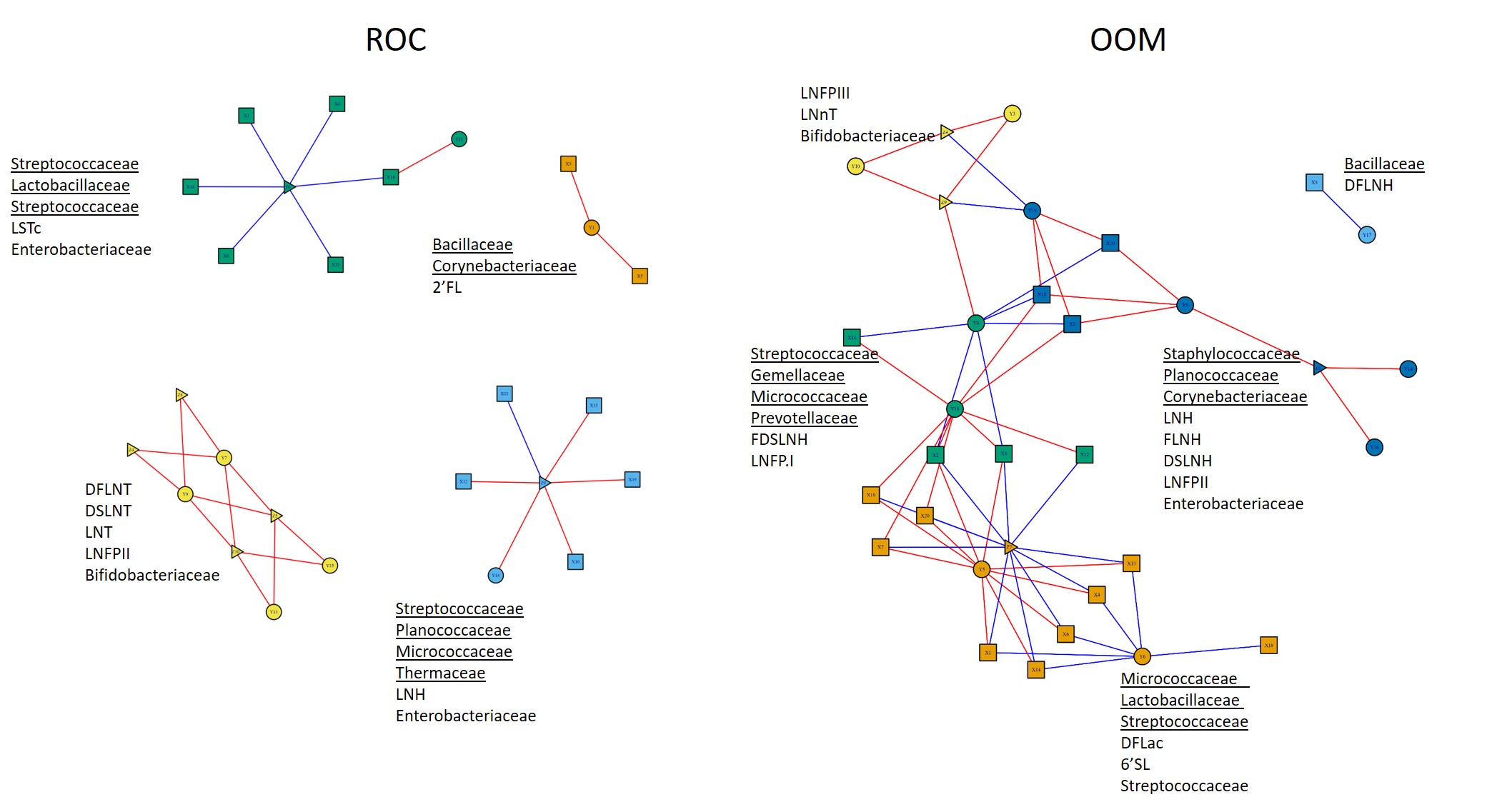

Supplement: Supplementary Figure 1 — Association between HMOs, human milk microbiome, and infant gut microbiome. Associations were measured using partial least square regression to identify communities represented by networks with blue, green, yellow, and gold node colors; using six Rochester mother-infant pairs and six OOM mother-infant pairs. Edges represent R 2 > 0.73 for ROC and 0.63 for OOM. Red and blue lines represent positive and negative correlations, respectively. The shapes of the nodes represent components: squares, human milk microbiota (underlined); circles, HMOs; triangles, infant stool microbiota. [file Image_1.jpeg]
