# Supplementary material for: Traditional Farming Lifestyle in Old Older Mennonites Modulates Human Milk Composition
Source: Front Immunol. 2021 Oct 11;12:741513. doi: 10.3389/fimmu.2021.741513 (PMC8545059; doi:10.3389/fimmu.2021.741513)
Supplement: Supplementary Table 2 — Human milk oligosaccharides measured. [file Table_2.docx]

Supplementary Table 2. Human milk oligosaccharides measured.

| Abbreviation | Name |
| --- | --- |
| 2'FL | 2'-fucosyllactose |
| 3'FL | 3'-fucosyllactose |
| LNnT | Lacto-N-neotetraose |
| 3'SL | 3'-sialyllactose |
| DFLac | Difucosyllactose |
| 6'SL | 6'-sialyllactose |
| LNT | Lacto-N-tetraose |
| LNFP I | Lacto-N-fucopentaose I |
| LNFP II | Lacto-N-fucopentaose II |
| LNFP III | Lacto-N-fucopentaose III |
| LSTb | LS-tetrasaccharide b |
| LSTc | LS-tetrasaccharide c |
| DFLNT | Difucosyllacto-N-tetraose |
| LNH | Lacto-N-hexaose |
| DSLNT | Disialyllacto-N-tetraose |
| FLNH | Fucosyllacto-N-hexaose |
| DFLNH | Difucosyllacto-N-hexaose |
| FDSLNH | Fucosyl-disialyllacto-N-hexaose |
| DSLNH | Disialyllacto-N-hexaose |
